# A Novel Cyclized Polyacrylonitrile Binder Strategy for Efficient Oxygen Evolution Reaction Catalysts

**DOI:** 10.3390/polym17182477

**Published:** 2025-09-13

**Authors:** Yifan Gu, Xiaomin Yin, Xinrong Li, Huili Ding, Xiaojie Zhang, Yi Feng

**Affiliations:** Hebei Key Laboratory of Functional Polymers, Department of Polymer Materials and Engineering, Hebei University of Technology, Tianjin 300130, China; gyftougaoyouxiang@163.com (Y.G.); ctst123xmyin@163.com (X.Y.); lixinrong1237@163.com (X.L.); aleeding@hebut.edu.cn (H.D.)

**Keywords:** cyclized polyacrylonitrile (CPAN), pyridinic nitrogen, binder, OER catalysts

## Abstract

In alkaline water electrolysis, conventional polymer binders like Nafion suffer from poor hydroxide conductivity and inadequate interfacial properties. Herein, a thermally cyclized polyacrylonitrile (CPAN) binder system with a conjugated ladder structure is introduced. The CPAN binders are synthesized by controlled thermal treatment under various temperatures, among which CPAN-400 demonstrates the optimal 57.03% pyridinic N content, provides π-conjugated pathways for enhanced electronic conductivity, and indicates hierarchically porous electrode architectures. The NiFe/CPAN-400 electrode achieves enhanced oxygen evolution performance with an overpotential of 354 mV at 100 mA cm^−2^, which is 153 mV and 103 mV lower than NiFe–Nafion and NiFe–PAN, respectively. This enhancement results from synergistic effects, including an electrochemically active surface area increased 2.3-fold, improved electrolyte wettability, and optimized charge transfer kinetics. The pyridinic nitrogen-enriched structure also facilitates a rate-determining step transition from charge transfer to *OOH formation, with a Tafel slope of 59.9 mV dec^−1^. This work establishes thermally induced polymer cyclization as a versatile strategy for advanced binder developments.

## 1. Introduction

The worldwide trend towards renewable energy has positioned hydrogen as a vital component in future energy systems, with water electrolysis emerging as the most viable approach to producing “green hydrogen” from clean electricity [1]. The performance of water electrolysis is markedly affected by the catalytic layers [2,3]. As a vital component of the catalytic layer, binders ensure mechanical stability, facilitate electron transport, and provide effective interfaces between catalysts, electrolytes, and gas phases [4,5,6,7,8]. Nafion is currently utilized as the predominant binder material in electrocatalytic systems; however, there are significant limitations when applied in alkaline oxygen evolution reaction (OER) environments [9]. The hydroxide ion transport is impeded by the perfluorosulfonic acid composition of the Nafion structure. Additionally, the hydrolysis of sulfonic groups in high-pH environments results in increased electrical resistance and decreased ion conductivity [10,11]. Recent studies indicate that binder systems can significantly improve electrochemical performance beyond merely offering structural support. The catechol groups in the bio-inspired polydopamine (PDA) coating exhibit metal-binding capabilities. When applied to electrode materials via dip-coating, it forms a hydrophilic and gas-venting surface, thereby enhancing the electrochemical performance of the electrode [12]. Also, the CoMoS_x_O_y_@PDA electrode exhibits superhydrophilicity (with a water contact angle of 0°) and superaerophobicity (with an underwater bubble contact angle of 16°) due to the abundance of strong hydrophilic groups such as phenolic hydroxyl and amino groups in PDA. This superhydrophilic/superaerophobic structure results in a low charge transfer resistance of 1.4 Ω and a low overpotential of 311 mV even at a high current density of 300 mA cm^−2^ [13]. Hydrogel polymers, such as poly(acrylic acid) (PAA), form robust and flexible networks that exhibit resistance to cracking via ionic crosslinking. Certain polymers also optimize the microenvironment of electrochemical procedures. The quaternary ammonium groups abundant in QPS provide a high-pH microenvironment, which boosts the activity of both the hydrogen evolution reaction (HER) and OER in anion exchange membrane water electrolysis (AEMWE). Furthermore, this ionomer is free from benzene structures, with which acidic phenolic compounds may form and lead to degradation of OER performance [14]. Conductive polymer composites, including PAA-polyaniline networks, provide abundant hydrophilic groups like -COOH and -OH, which form 3D networks via hydrogen bonding. The 3D network significantly enhances the binding strength to silicon particles and the current collector. As a result, silicon anodes incorporating this binder demonstrate excellent electrochemical performance [15]. However, notable limitations such as inadequate charge transfer and mass transfer, as well as unsatisfactory chemical stability, constitute a significant barrier to the advancement of water electrolysis technology for industrial applications.

Polyacrylonitrile (PAN) exhibits a unique thermal transformation property for modifying its molecular structure [16]. Controlled heating (180–300 °C) of PAN induces dehydrogenation and cyclization, leading to the formation of ring structures from nitrile groups and the development of ladder-like polymer chains. Heating at temperatures between 300 and 500 °C promotes aromatization, leading to the development of nitrogen-rich domains within conductive carbon-like networks [17,18,19]. This thermal transformation produces a multifunctional cyclized PAN (noted as CPAN) that demonstrates remarkably enhanced electrical conductivity, strong affinity with catalyst particles, and a porous catalyst–binder structure that facilitates the transport of both electrolyte and gas. Previous studies on batteries indicate that cyclized PAN maintains 83% of its performance after 800 cycles; however, its potential use in alkaline OER remains unexplored [20].

## 2. Experimental Section

### 2.1. Materials

All reagents were utilized without further purification: nickel(II) nitrate hexahydrate (Ni(NO_3_)_2_·6H_2_O, 98.5%, Macklin, Shanghai, China), iron(III) nitrate nonahydrate (Fe(NO_3_)_3_·9H_2_O, 98.5%, Macklin), urea (98%, Bide Pharmatech, Shanghai, China), ammonium fluoride (NH_4_F, 96%, Aladdin, Shanghai, China), polyacrylonitrile (PAN, Mₙ = 150,000, Macklin), N,N-dimethylformamide (DMF, 99.8%, Macklin), potassium hydroxide (KOH, 95.0%, Macklin), Nafion^®^ solution (5 wt%, D520, Adamas), ultrapure water (18.25 MΩ·cm), carbon cloth (Zhengtaitong, Suzhou, China), absolute ethanol (99.5%, Guangshunda, Tianjin, China), and isopropanol (99.7%, Macklin).

### 2.2. Synthesis of NiFe Alloy Catalyst

**Scheme 1:**
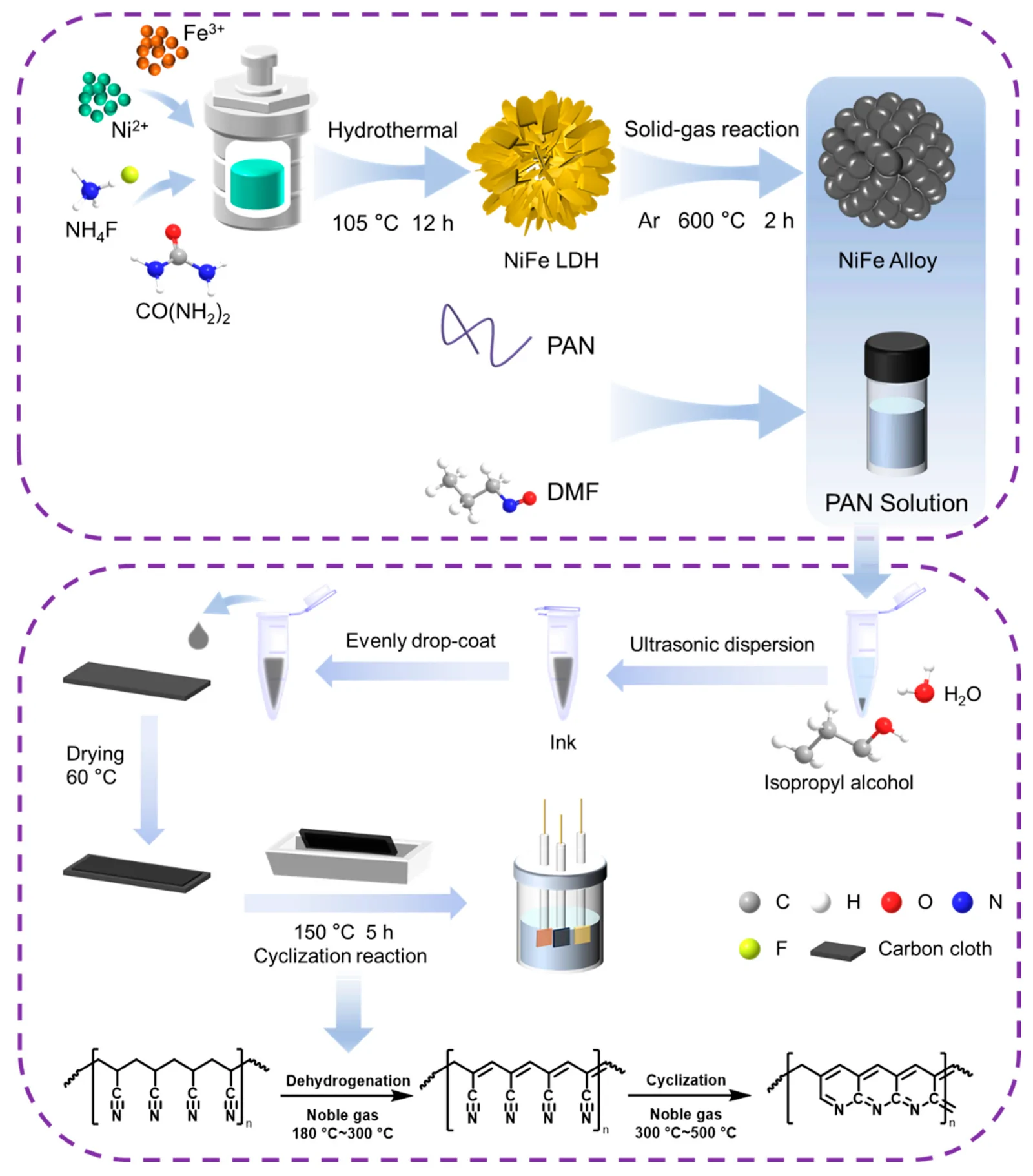
Illustration of experimental process.

The NiFe alloy catalyst was synthesized via a hydrothermal-reduction methodology: initially, 1.8 mmol Ni(NO_3_)_2_·6H_2_O, 0.9 mmol Fe(NO_3_)_3_·9H_2_O, 64 mmol urea, and 13.5 mmol NH_4_F were dissolved in 50 mL ultrapure water under magnetic stirring (550 rpm) for 1 h at ambient temperature (25 °C) to obtain a homogeneous bluish-green precursor solution. The resulting mixture was subsequently transferred to a 100 mL Teflon-lined stainless-steel autoclave and subjected to hydrothermal treatment at 105 °C for 12 h. The obtained NiFe layered double hydroxide (LDH) precursor was thoroughly washed with DI water and ethanol, collected by vacuum filtration, and dried at 60 °C overnight under vacuum. The dried precursor was then reduced under a H_2_/Ar atmosphere (5 vol% H_2_) in a tube furnace with a controlled heating rate of 5 °C min^−1^ to 600 °C, followed by isothermal annealing for 2 h, yielding the black NiFe alloy catalyst (Scheme 1).

### 2.3. Preparation of the Electrodes

Pretreated carbon cloth substrates (1 cm × 3 cm) were prepared by sequential acid washing and vacuum drying to remove surface contaminants and enhance wettability. The catalyst ink was formulated by dispersing 5 mg of the as-synthesized NiFe alloy in 250 μL isopropanol via ultrasonication, followed by the sequential addition of 200 μL ultrapure water and 50 μL PAN solution (5 wt% in DMF), with subsequent ultrasonication for 30 min to ensure homogeneous dispersion. A volume of 100 μL catalyst ink was uniformly drop-cast onto the pretreated carbon cloth substrate and vacuum-dried at 60 °C. The electrodes were then subjected to thermal cyclization under an inert Ar atmosphere at various target temperatures (150–500 °C) with a controlled heating rate of 5 °C min^−1^ and an isothermal holding period of 5 h, yielding the NiFe/CPAN-X electrodes (where X denotes the cyclization temperature). Control electrodes included NiFe/Nafion (wherein PAN was substituted with Nafion^®^) and NiFe/PAN (without thermal treatment). (Scheme 1)

### 2.4. Material Characterization

Crystallographic analysis was performed using powder X-ray diffraction (XRD, D8 Discover, Bruker, Rheinstetten, Germany) with Cu Kα radiation over a 2θ range of 10–90° at a scan rate of 5° min^−1^. Chemical bonding characteristics were investigated by Fourier-transform infrared spectroscopy (FT-IR, TENSOR 27, Bruker, Rheinstetten, Germany) using KBr pellets in the wavenumber range of 400–4000 cm^−1^. Surface chemical composition and electronic states were analyzed via X-ray photoelectron spectroscopy (XPS, ESCALAB 250Xi, Thermo Fisher, Waltham, MA, USA) employing Al Kα radiation with a pass energy of 30 eV. Morphological features were examined using scanning electron microscopy (SEM, Nova NanoSEM 450, FEI, Hillsboro, OR, USA) and transmission electron microscopy (TEM, JEM-2100F, JEOL, Tokyo, Japan) with samples prepared by ethanol dispersion on copper grids. Surface wettability was quantified through contact angle measurements.

### 2.5. Electrochemical Measurements

All electrochemical evaluations were conducted using a CHI 760E (Chenhua, Shanghai, China) electrochemical workstation in a conventional three-electrode configuration with 1 M KOH electrolyte, Hg/HgO reference electrode, and platinum wire counter electrode. Prior to measurements, electrodes were electrochemically activated via chronoamperometry to achieve steady-state performance. Linear sweep voltammetry (LSV) was performed at a scan rate of 2 mV s^−1^ with iR compensation to determine electrocatalytic activity. Tafel slopes were extracted from the linear region of the Tafel plots according to the relationship η = a + b log|j|, where η represents overpotential, j denotes current density, and b is the Tafel slope. Electrochemical impedance spectroscopy (EIS) was conducted over a frequency range of 0.1–100,000 Hz with an AC amplitude of 5 mV, and the potential was 1.67 V vs. RHE. Electrochemically active surface area (ECSA) was determined from the double-layer capacitance derived from cyclic voltammetry scans at varying scan rates (20–100 mV s^−1^) within the non-Faradaic potential window (0.01–0.11 V vs. Hg/HgO). Long-term stability was evaluated via chronoamperometry at a constant current density of 50 mA cm^−2^. All potentials were converted to the reversible hydrogen electrode (RHE) scale using the Nernst equation:

$$E_{\mathrm{RHE}\;}=\;E_{\mathrm{Hg}/\mathrm{HgO}}\;+\;0.059\;\text{×}\;\mathrm{pH}\;+\;0.098$$

## 3. Results and Discussion

A crystalline NiFe alloy electrocatalyst was effectively produced using a hydrothermal-reduction method. XRD investigation validated the synthesis of crystalline NiFe LDH precursors (PDF#49-0188, Figure S1), exhibiting distinctive diffraction peaks at 11.8° (003), 23.4° (006), and 34.6° (012), in accordance with the hexagonal crystal structure [21]. After thermal reduction in a H_2_/Ar environment, NiFe alloy catalysts were obtained (JCPDS PDF#47-1405), displaying significant peaks at 43.6° (111), 50.8° (200), and 74.7° (220), thereby validating the successful synthesis of the face-centered cubic metallic phase [22]. The temperature-dependent structural transformation of PAN in the production of NiFe-CPAN electrodes entails sequential dehydrogenation, cyclization, aromatization, oxidation, and crosslinking reactions that collectively enable the formation of conjugated ladder structures of CPAN. Pristine PAN displays distinct diffraction peaks at 16.82° and 29.41° (Figure S2), signifying semicrystalline polymer domains. The peak positions and intensities remain mostly unchanged after heat treatment at 150 °C and 200 °C, indicating negligible structural rearrangement within the polymer matrix. Thermal processing at 400 °C induces considerable peak intensity reduction, indicating disruption of the native polymer structure. A prominent diffraction peak appears at 25.79° for the material treated at 500 °C, matching the (002) crystallographic plane of quasi-graphitic carbon domains, indicative of efficient carbonization processes [23,24].

**Figure 1:**
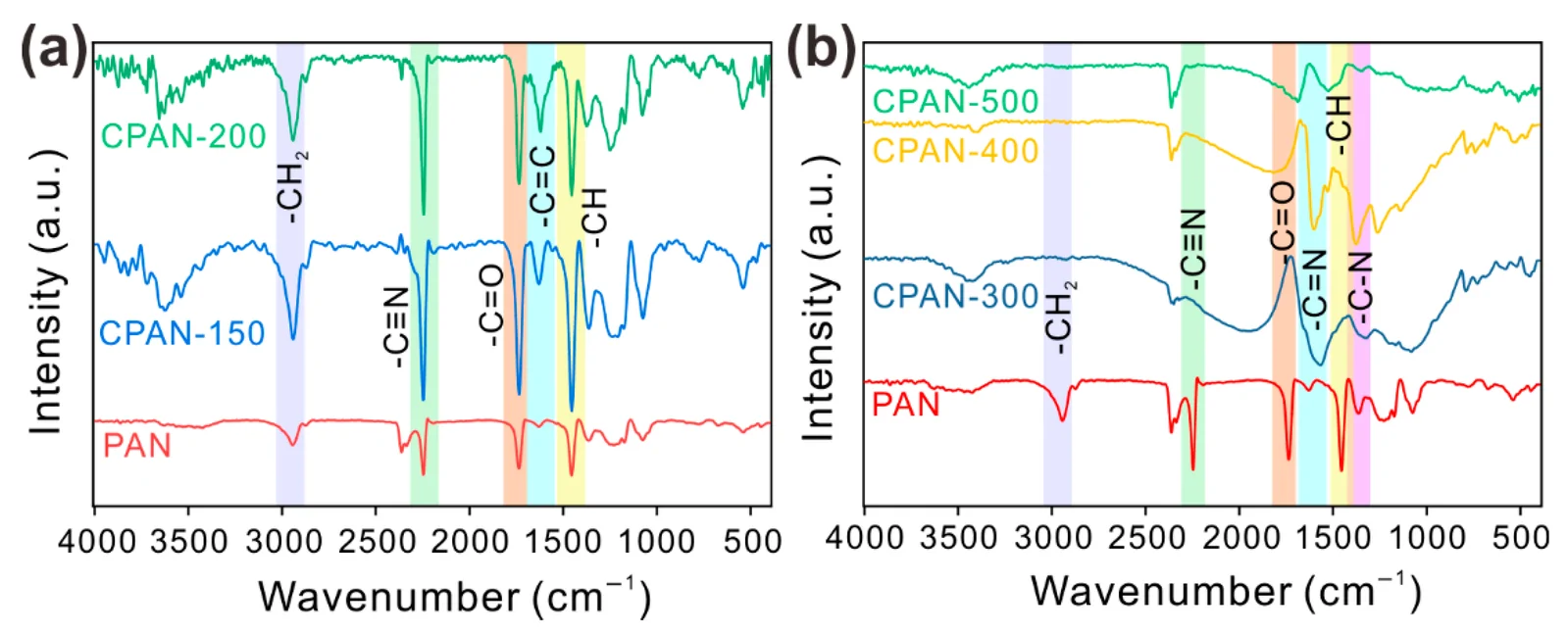
(**a**,**b**) FTIR spectra of PAN, CPAN-150, CPAN-200, CPAN-300, CPAN-400, and CPAN-500.

FT-IR (Figure 1) provides molecular-level insights into the chemical bonding evolution during thermal treatment. Pristine PAN displays characteristic nitrile (-C≡N) stretching vibrations at 2243.1 cm^−1^, accompanied by aliphatic C-H stretching modes at 2943.2 cm^−1^ and 1457.5 cm^−1^ [25]. Upon thermal treatment at 150–200 °C, emergent C=C stretching vibrations appear at 1618 cm^−1^, confirming the initiation of dehydrogenation reactions without extensive cyclization. At elevated temperatures (300 °C), progressive attenuation of –C≡N peaks occurs concomitantly with the emergence of C=N (1566.1 cm^−1^) and C–N (1362.2 cm^−1^) vibrational modes, signifying cyclization into thermally stable conjugated ladder polymers [25,26,27]. This systematic structural evolution from linear polymer chains to conjugated heterocyclic networks significantly enhances electronic conductivity and interfacial stability, properties that are critical for high-performance electrocatalytic applications.

**Figure 2:**
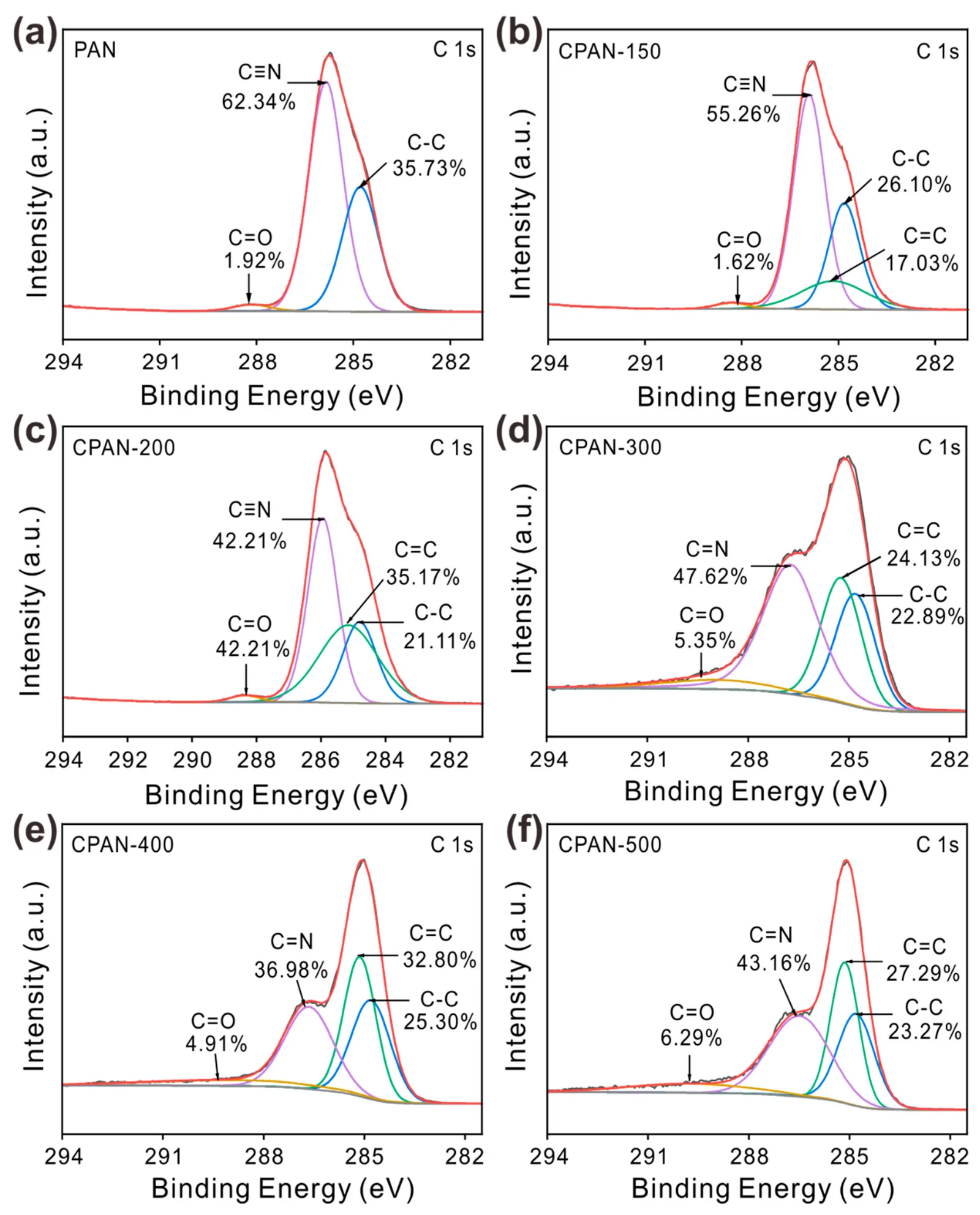
High resolution XPS spectra of C 1s (**a**) PAN, (**b**) CPAN-150, (**c**) CPAN-200, (**d**) CPAN-300, (**e**) CPAN-400, and (**f**) CPAN-500.

**Figure 3:**
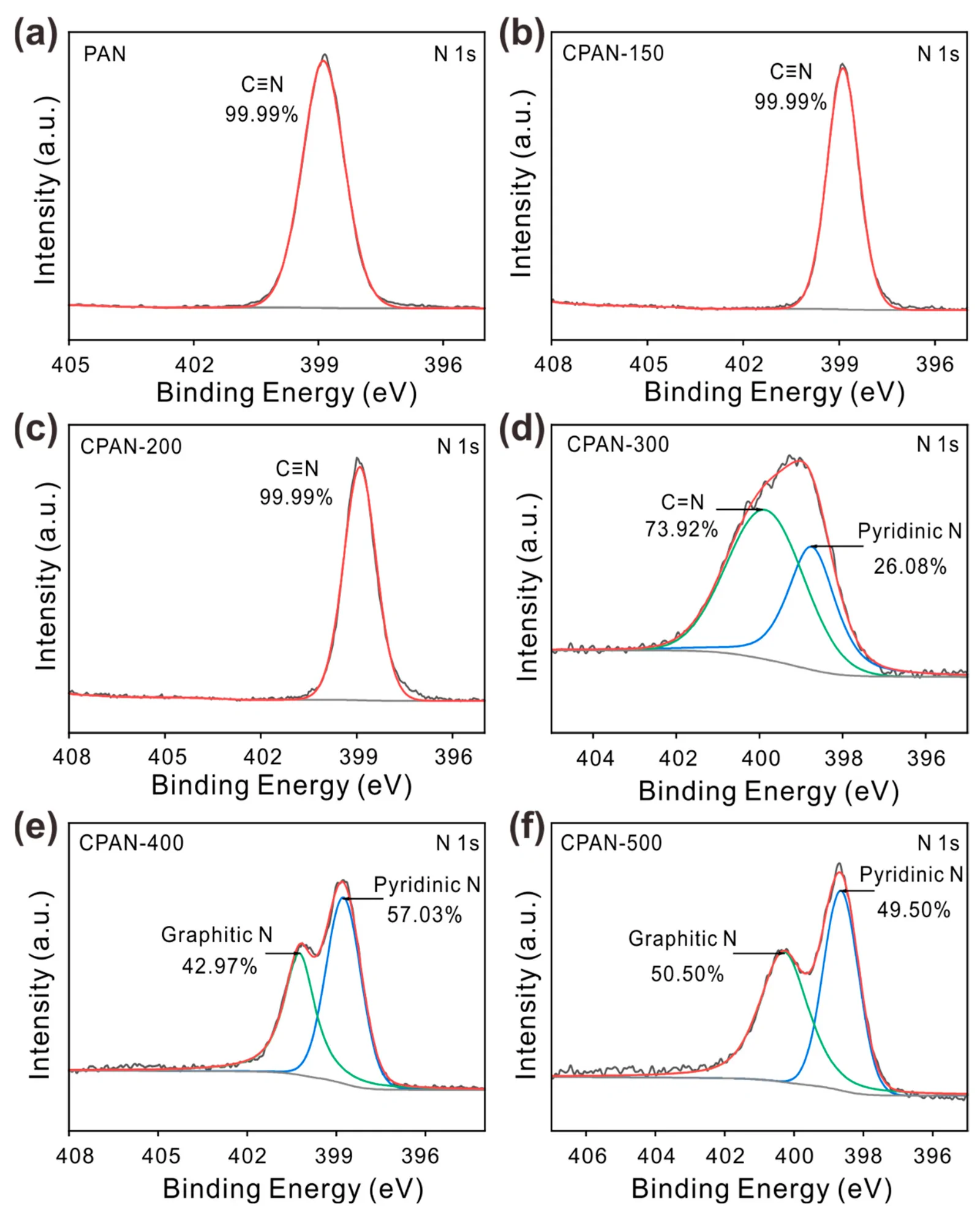
High resolution XPS spectra of N1s (**a**) PAN, (**b**) CPAN-150, (**c**) CPAN-200, (**d**) CPAN-300, (**e**) CPAN-400, and (**f**) CPAN-500.

XPS analysis indicates a temperature-dependent structural evolution of PAN-derived binders, which is directly associated with progressive cyclization and graphitization mechanisms (Figure 2, Figure 3, and Figure S3). In the C 1s spectra (Figure 2a–f), predominant C-C bonds at 284.8 eV and minor C=O/O-C=O moieties at 288.7 eV (attributed to ambient oxidation) can be observed in pristine PAN and CPAN samples obtained under various temperatures [28,29]. Pristine PAN exhibits a significant -C≡N peak at 286.2 eV [19], accounting for an area fraction of 62.34%. In CPAN-150, the area fraction of this peak decreases to 55.26% and further to 42.21% in CPAN-200, alongside that of C=C bonds (285.2 eV) [23], which increases from 17.03% to 35.17%. This observation confirms a dehydrogenation-driven chain reorganization. At a treating temperature of 300 °C, the -C≡N signal disappears, being replaced by C=N bonds (286.2 eV) [30] in CPAN-300/400/500, indicating the formation of a heterocyclic ladder structure. Additional evidence for this transformation is provided by N 1s spectra (Figure 3a–f). -C≡N (398.6 eV) [31] can be observed in CPAN-200 samples, whereas pyridinic N (398.8 eV) [32] is detected in CPAN-300, CPAN-400, and CPAN-500 samples. The area fraction of pyridinic N increases from 26.08% at 300 °C to a peak of 57.03% at 400 °C, suggesting optimal cyclization. At 500 °C, the proportion of pyridinic nitrogen decreases to 49.5%, while graphitic nitrogen emerges at 400.1 eV [31], indicating the carbonization via dehydrogenative condensation and aromatic stacking [30,33,34,35]. The 57.03% area fraction of pyridinic N at 400 °C indicates an optimal balance between conductivity enhancement due to cyclization and the maintenance of nitrogen functionalities that support charge transfer at the catalyst–binder interface. The refinement of the electronic structure would boost the OER kinetics and promote the electrocatalytic performance, since the pyridinic N facilitates charge delocalization in the conjugated ladder structure [36,37], while ensuring interfacial compatibility with the NiFe catalyst. The synergistic effect of optimized nitrogen species and the formation of a conjugated network establish the mechanistic basis for the enhanced OER kinetics seen in the cyclized binder system, highlighting the essential role of precise thermal treatment in binder optimization strategies.

**Figure 4:**
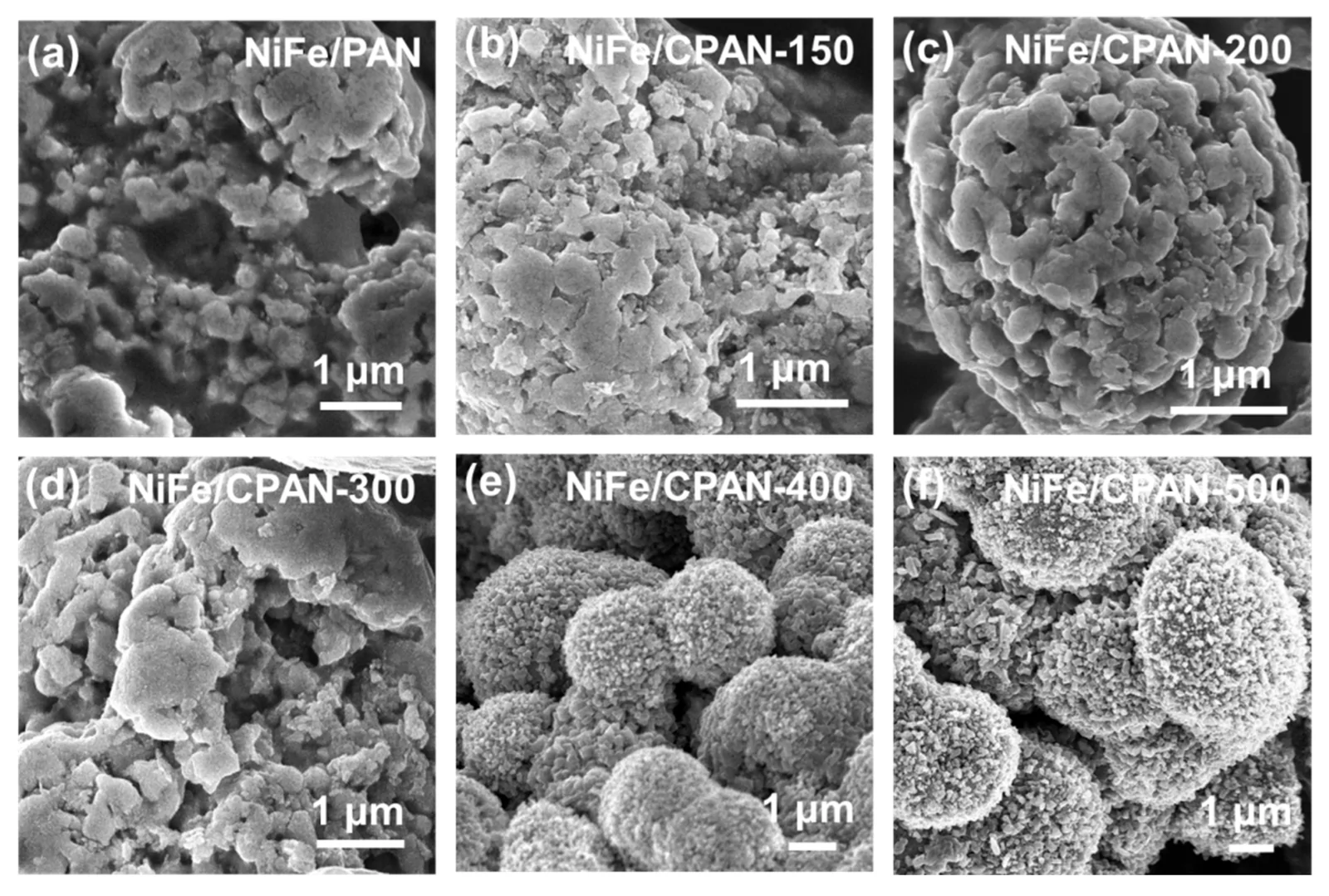
SEM images of the synthesized PAN based electrodes: (**a**) NiFe/PAN, (**b**) NiFe/CPAN-150, (**c**) NiFe/CPAN-200, (**d**) NiFe/CPAN-300, (**e**) NiFe/CPAN-400, and (**f**) NiFe/CPAN-500.

**Figure 5:**
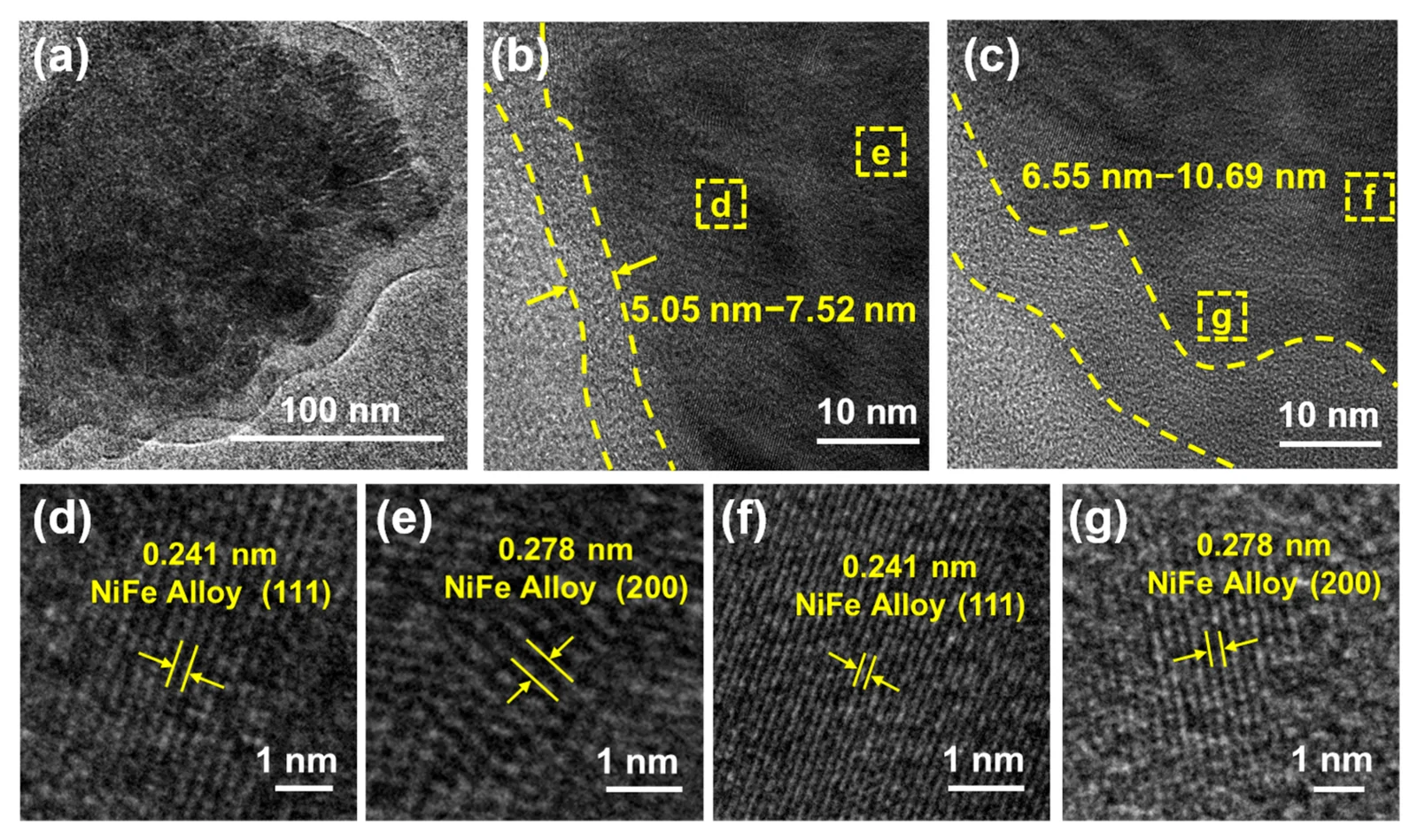
NiFe/CPAN-400 of (**a**) TEM and (**b**,**c**) HRTEM images; (**d**–**g**) magnified images of the crystalline regions.

The morphology evolution of synthesized NiFe-based electrodes due to thermal cyclization was systematically investigated via SEM. As depicted in Figure 4a and Figure S4, both NiFe/Nafion and NiFe/PAN electrodes exhibit dense polymer agglomerates that extensively encapsulate the catalyst particles, resulting in limited active site accessibility and porosity. Similar morphologies can be observed in NiFe/CPAN-150 and NiFe CPAN-200. Upon thermal treatment at temperatures above 300 °C, the in situ cyclization process transforms the electrode architecture. Although not obvious in the SEM image of NiFe/CPAN-300 (Figure 4d), thermal-induced cyclization generates conformal CPAN coatings that effectively eliminate polymer aggregation, leading to the formation of hierarchically porous networks (Figure 4e,f). Notably, the electrode processed at 400 °C and 500 °C demonstrates porous nanoballs assembled by densely distributed catalyst nanoparticles. The hierarchical porosity may maximize the electrochemical interface, which facilitates efficient electrolyte penetration and gas transport, and represents a substantial improvement compared with conventional polymer binder systems that typically yield non-porous architectures. TEM further provides critical insights into the nanoscale architecture of the cyclized NiFe-CPAN-400. In Figure 5a, conformal CPAN coating, which uniformly encapsulates the NiFe alloy catalysts, can be observed. Further high-resolution TEM images (Figure 5b,c) reveal the thickness of amorphous CPAN coating ranging from 5 to 11 nm. The crystalline nature of the NiFe cores is confirmed by distinct lattice fringes corresponding to d-spacings of 0.241 nm (111) and 0.278 nm (200), indicating the preservation of the alloy structure during the cyclization process [22]. The intimate interface between the crystalline NiFe cores and the amorphous CPAN overlayer creates a multifunctional nano-interface. According to XPS analysis, the in situ-formed CPAN coating layer derives from extended π-conjugation through C=N bond formation, which facilitates efficient electron transfer to active sites. Simultaneously, the ladder polymer structure provides mechanical stabilization of the catalyst-electrode interface, effectively preventing delamination during vigorous oxygen bubble evolution [20,38,39].

**Figure 6:**
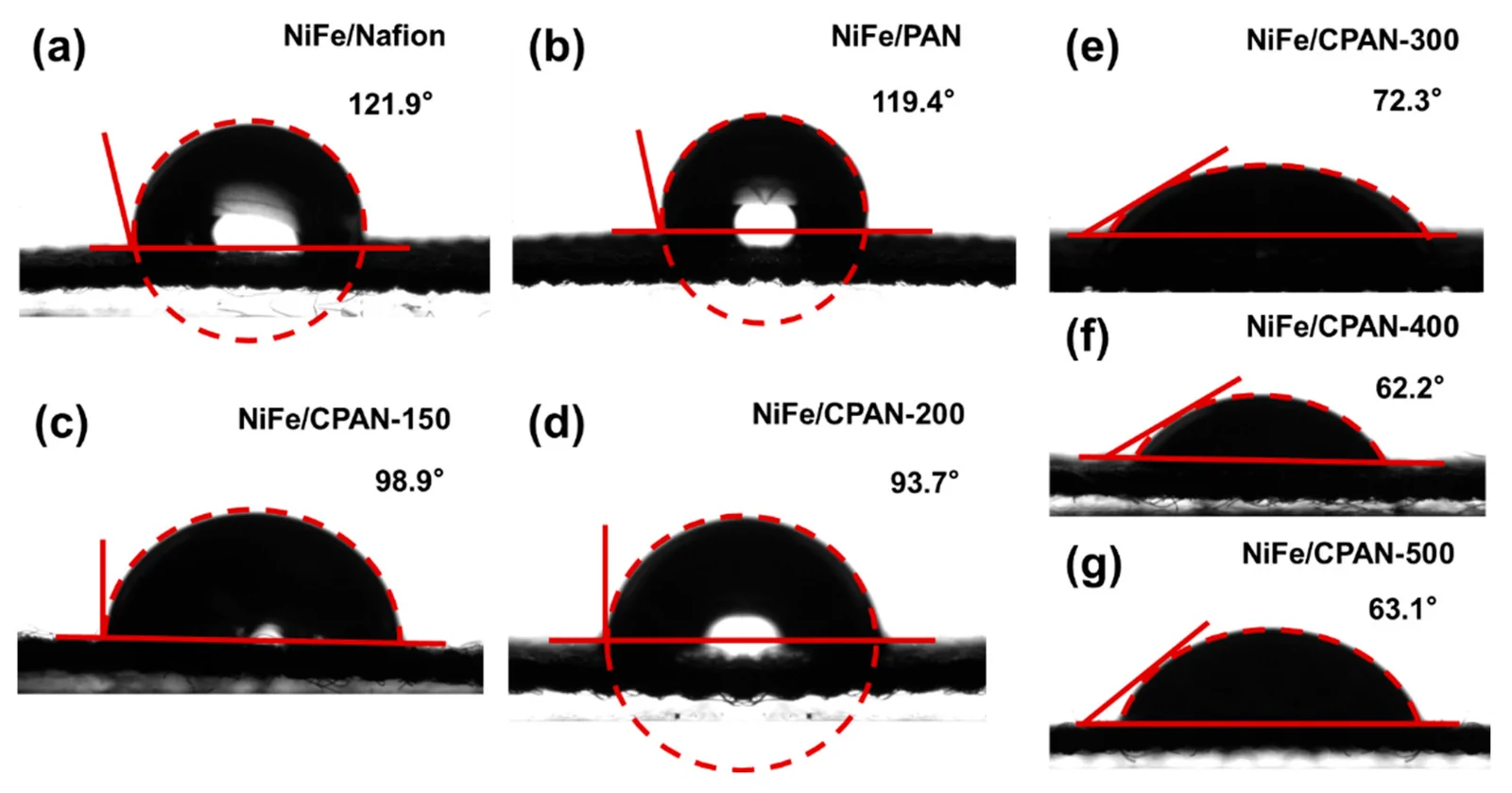
Contact angle between electrodes and water: (**a**) NiFe/Nafion, (**b**) NiFe/PAN, (**c**) NiFe/CPAN-150, (**d**) NiFe/CPAN-200, (**e**) NiFe/CPAN-300, (**f**) NiFe/CPAN-400, and (**g**) NiFe/CPAN-500.

The transformative effect of CPAN cyclization on electrolyte wetting behavior was quantified using contact angle measurements. The NiFe/CPAN-400 electrode achieves an optimized contact angle of 62.2°, which is a significant enhancement of 59.7° and 57.2° compared to NiFe/Nafion (121.9°) and untreated NiFe/PAN (119.4°), respectively (Figure 6a–g) [40]. This improved hydrophilicity facilitates a more thorough infiltration of the electrolyte into the hierarchical structure and facilitates the rapid detachment of bubbles at triple-phase boundaries [41,42]. The modest rebound observed at 500 °C (63.1°) is a confirmation that 400 °C is the optimal processing temperature, as it balances hydrophilicity and conductivity before excessive graphitization compromises surface polarity [43].

**Figure 7:**
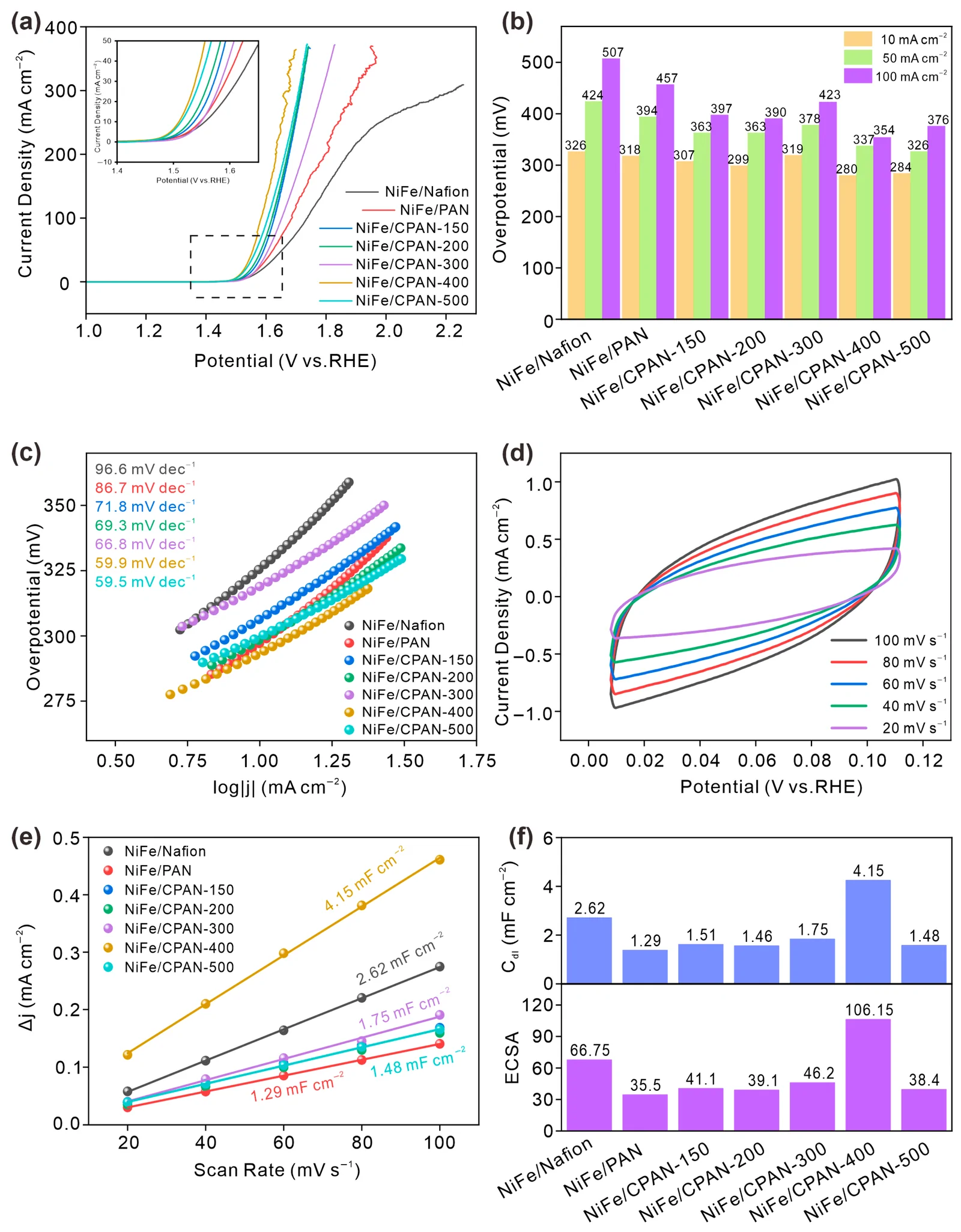
Electrocatalytic activities of the synthesized Nafion and PAN based electrodes for OER: (**a**) LSV polarization curves; (**b**) comparison of the overpotentials required at 10 mA cm^−2^, 50 mA cm^−2^ and 100 mA cm^−2^, respectively; (**c**) corresponding Tafel plots; (**d**) CV curves under various scan rates (scan rates: 20 to 100 mV s^−1^) of NiFe/CPAN-400; (**e**) C_dl_ determined by the CV curves under various scan rates (scan rates: 20 to 100 mV s^−1^); and (**f**) a comparison of C_dl_ and ECSA of the synthesized electrode materials.

The OER performance of synthesized NiFe-based electrodes was evaluated via LSV measurements. It demonstrates that the NiFe/CPAN-400 electrode achieves exceptional OER performance with a low overpotential of 354 mV at 100 mA cm^−2^ (Figure 7a,b), which represents significant improvements of 153 mV and 103 mV compared to NiFe/Nafion (507 mV) and NiFe/PAN (457 mV), respectively. Among NiFe/CPAN electrodes synthesized under various temperatures, the electrochemical performance of NiFe/CPAN-400 surpasses its counterparts which exhibit higher overpotential at 10 mA cm^−2^, 50 mA cm^−2^ and 100 mA cm^−2^. The Tafel slope analysis further confirms the accelerated OER kinetics, with NiFe/CPAN-400 exhibiting a slope of 59.92 mV dec^−1^, substantially lower than its counterparts (86.7–96.6 mV dec^−1^, Figure 7c). This near-theoretical Tafel slope suggests that the rate-determining step transitions from charge transfer to the formation of *O from *OH, according to the adsorbent evolution mechanism (AEM) [44], indicative of highly efficient catalytic kinetics.

**Figure 8:**
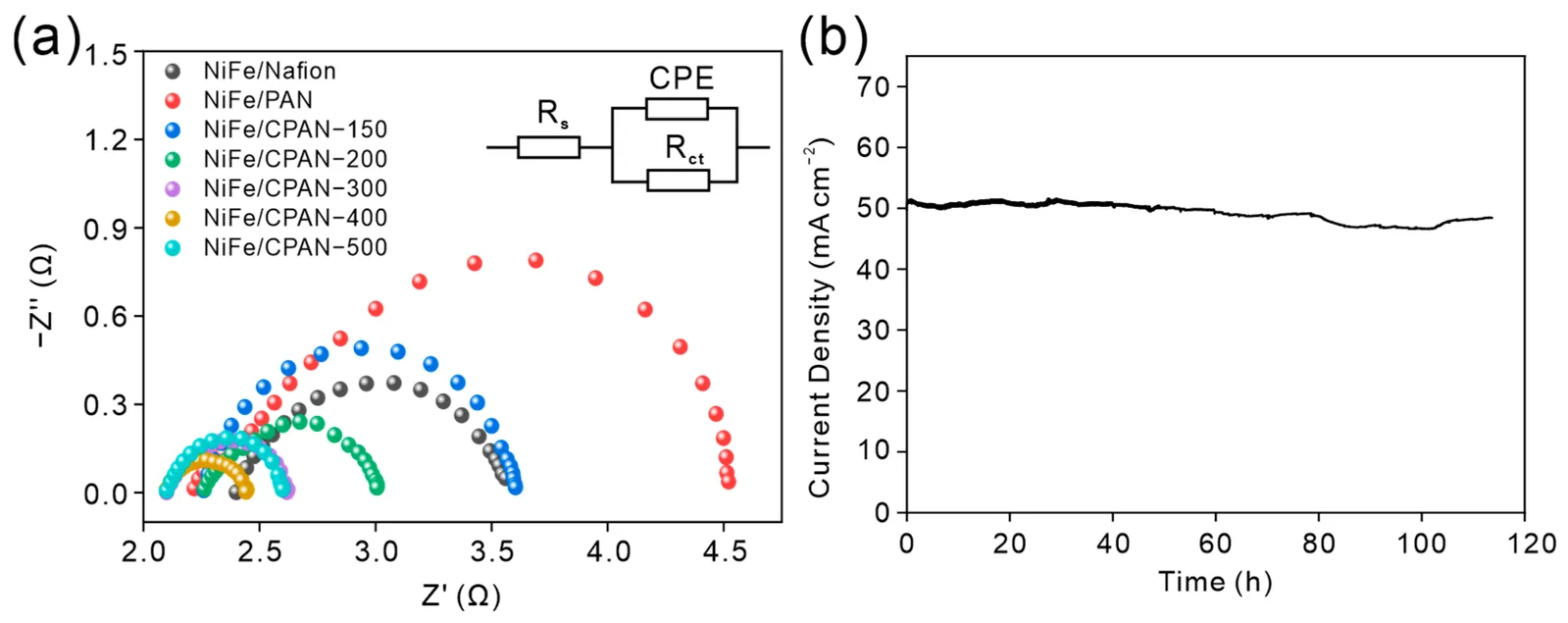
(**a**) Nyquist plots of the synthesized electrode materials and (**b**) the stability curve of NiFe/CPAN-400.

The electrochemically active surface area (ECSA) was ascertained by examining the double-layer capacitance (C_dl_) through cyclic voltammetry (CV) measurements (Figure 7d), which revealed substantial improvements in active site accessibility. The NiFe/CPAN-400 electrode exhibits a C_dl_ of 4.15 mF cm^−2^, which corresponds to an ECSA of 106.15 cm^2^ (Figure 7e,f). This is a 2.3-fold enhancement compared to Nafion-based electrodes, demonstrating that the hierarchically porous electrode architecture induced by CPAN cyclization has resulted in more exposed active sites for OER. Further, the charge transfer capability of the electrodes was evaluated by electrochemical impedance spectroscopy (EIS) analysis (Figure 8a). The NiFe/CPAN-400 system exhibits the minimal charge transfer resistance (R_ct_ = 0.34 Ω) among NiFe-CPAN counterparts, NiFe–PAN and NiFe–Nafion configurations (Table S1), and it also has excellent long-term stability (Figure 8b). The remarkable charge transfer optimization is believed to stem from the pyridinic N that promotes charge delocalization within the C=N bond and the π-conjugated ladder structure of CPAN, which correlates with the analysis of Tafel slope that the rate-determining step has moved from charge transfer to the formation of *OOH [45].

Based on a thorough electrochemical analysis that revealed significantly improved active site accessibility and charge transfer kinetics, we attribute the promoted electrocatalytic performance of NiFe/CPAN-400 to the unique conjugated ladder structure with optimized electronic and mass transport processes. Firstly, the 57.03% pyridinic N in CPAN-400 (XPS, Figure 3) creates extended π-conjugated networks that facilitate rapid electron transfer to NiFe active sites [46]. Moreover, the pyridinic N is reported to be beneficial to optimize the d-band center of NiFe alloy [47] that lowers the energy barrier of *O to *OOH, which accelerates the OER kinetics [48]. In addition, the CPAN binder with conjugated ladder structure improves the hydrophilicity, which enables complete electrolyte infiltration, and the hierarchical porous structure of NiFe/CPAN benefits the O_2_ bubble dissipation, which decreases the mass-transfer limitations.

## 4. Conclusions

In this work, thermally cyclized polyacrylonitrile (CPAN) binders have been applied to NiFe alloy electrocatalysts for efficient alkaline OER. The controlled thermal treatment of PAN at 400 °C triggers structural transformation from linear polymer chains to conjugated ladder networks with optimal 57.03% pyridinic nitrogen content, creating extended π-conjugated networks that facilitate efficient electron transfer while transforming the electrode morphology from dense polymer agglomerates to hierarchically porous structures. This cyclization process dramatically improves active site accessibility (ECSA of 106.25 cm^2^), electrolyte infiltration (contact angle reduced from 121.9° to 62.2°), and charge transfer kinetics (R_ct_ = 0.34 Ω), collectively resulting in exceptional OER performance with the NiFe/CPAN-400 electrode achieving a low overpotential of 354 mV at 100 mA cm^−2^ and a Tafel slope of 59.92 mV dec^−1^. The mechanistic insights reveal that the rate-determining step transitions from charge transfer to *OOH formation, indicating highly efficient catalytic kinetics enabled by the pyridinic nitrogen-enriched conjugated structure. This work establishes a new paradigm for binder design in electrocatalytic systems, demonstrating that strategic polymer modification can simultaneously address multiple performance-limiting factors, including electronic conductivity, mass transport, and interfacial compatibility. The thermally induced cyclization strategy offers a scalable and cost-effective approach for developing advanced binder materials, potentially accelerating the deployment of efficient water electrolysis technologies for sustainable hydrogen production and providing new insights for future investigations into structure–performance relationships in electrocatalytic applications.

## Data Availability

The original contributions presented in this study are included in the article/supplementary material. Further inquiries can be directed to the corresponding authors.

## Supplementary Materials

The following supporting information can be downloaded at: https://www.mdpi.com/article/10.3390/polym17182477/s1, Figure S1: XRD patterns of NiFe LDH and NiFe Alloy; Figure S2. XRD patterns of (a) CPAN-150, CPAN-200, and (b) PAN, CPAN-300, CPAN-400, and CPAN-500; Figure S3. High resolution XPS spectra of O 1s (a) PAN, (b) CPAN-150, (c) CPAN-200, (d) CPAN-300, (e) CPAN-400, and (f) CPAN-500; Figure S4. SEM images of the synthesized Nafion based electrodes; Table S1. All the electrochemical parameters of the prepared composite electrodes; Figure S5. CV curves under various scan rates (scan rates: 20 to 100 mV s^−1^) of the synthesized electrode materials: (a) NiFe/Nafion, (b) NiFe/PAN, (c) NiFe/CPAN-150, (d) NiFe/CPAN-200, (e) NiFe/CPAN-300, and (f) NiFe/CPAN-500; Table S2. Comparison of overpotentials for OER catalysts in the literature and in this work. References [47,48,49,50,51,52,53,54] are cited in the Supplementary Materials.
