# Supplementary material for: A Novel Cyclized Polyacrylonitrile Binder Strategy for Efficient Oxygen Evolution Reaction Catalysts
Source: Polymers (Basel). 2025 Sep 13;17(18):2477. doi: 10.3390/polym17182477 (PMC12473700; doi:10.3390/polym17182477)
Supplement: Supplementary file 1 [file polymers-17-02477-s001.zip › polymers-3854448-supplementary.pdf]

A Novel Cyclized Polyacrylonitrile Binder Strategy for Efficient Oxygen Evolution Reaction Catalysts

Yifan Gu[1,†], Xiaomin Yin[1,†], Xinrong Li[1], Huili Ding[1], Xiaojie Zhang[1,*] and Yi Feng[1,*]

[1] Hebei Key Laboratory of Functional Polymers, Department of Polymer Materials and Engineering, Hebei University of Technology, Tianjin 300130, P. R. China

[†] Yifan Gu and Xiaomin Yin contributes equally to this work

* E-mail: zhangxj@hebut.edu.cn (X. Zhang); luckyii0512@hebut.edu.cn (Y. Feng).

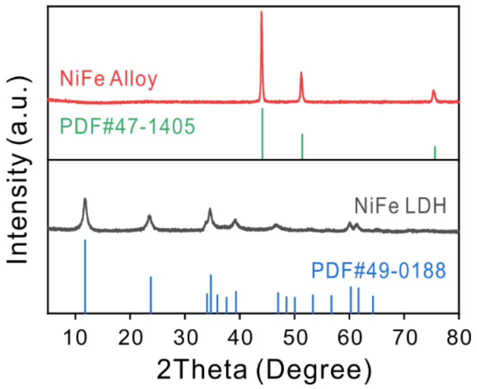

Figure S1. XRD patterns of NiFe LDH and NiFe Alloy.

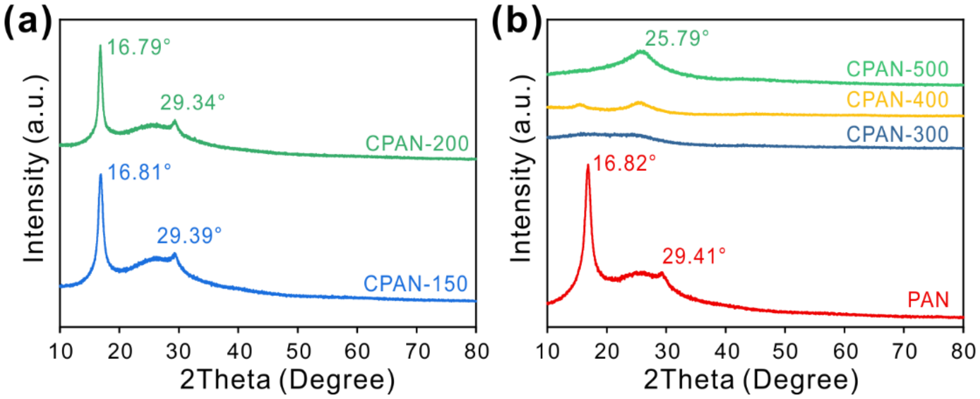

Figure S2. XRD patterns of (a) CPAN-150, CPAN-200, and (b) PAN, CPAN-300, CPAN-400, and CPAN-500.

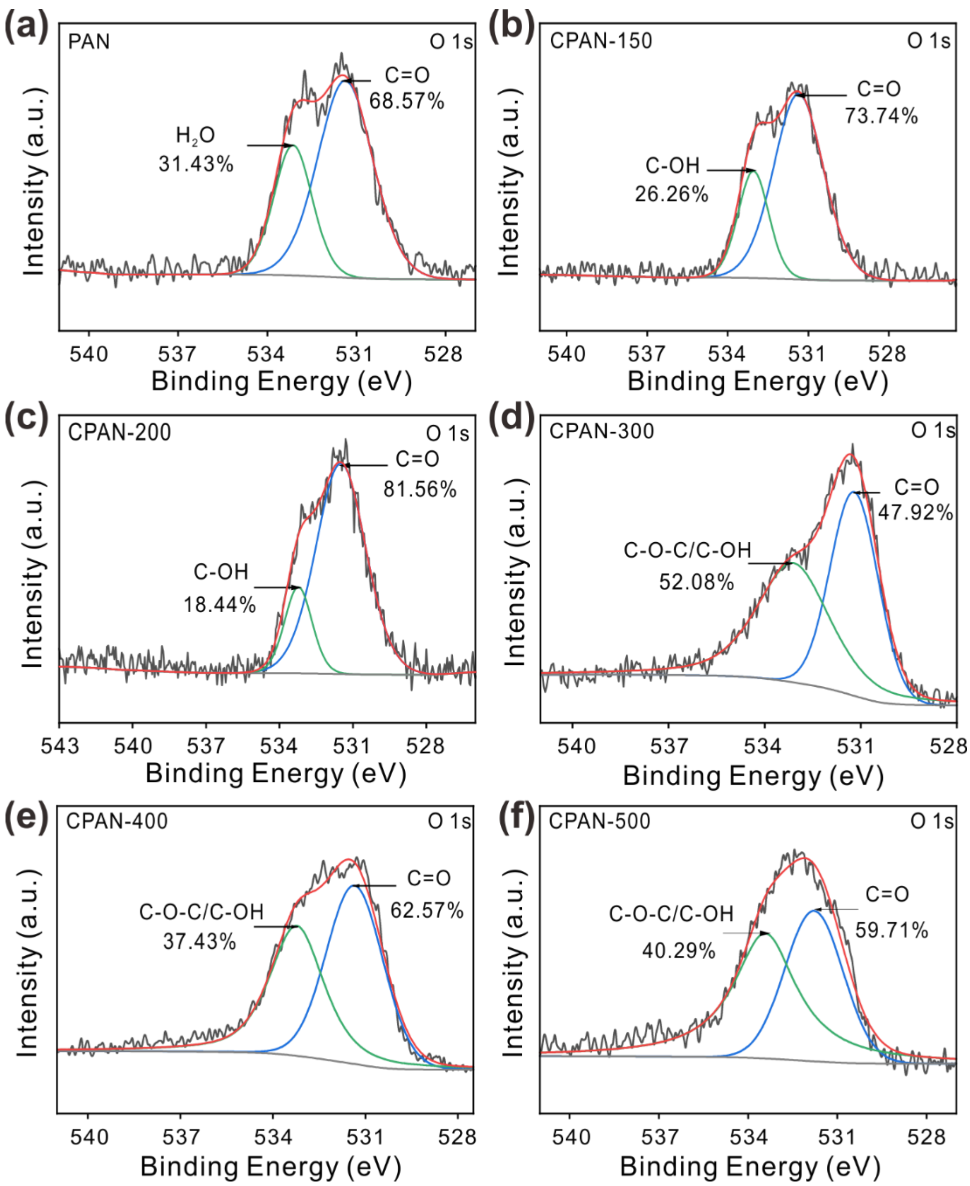

Figure S3. High resolution XPS spectra of O 1s (a) PAN, (b) CPAN-150, (c) CPAN-200, (d) CPAN-300, (e) CPAN-400, and (f) CPAN-500.

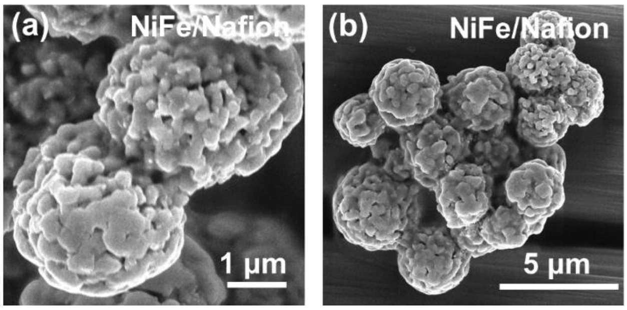

Figure S4. SEM images of the synthesized Nafion based electrodes.

Table S1. All the electrochemical parameters of the prepared composite electrodes

	NiFe/ Nafion	NiFe /PAN	NiFe/ CPAN-150	NiFe/ CPAN-200	NiFe/ CPAN-300	NiFe/ CPAN-400	NiFe/ CPAN-500
**R_{ct} (Ω)**	1.16	2.30	1.34	0.74	0.55	0.34	0.59
**R_s (Ω)**	2.40	2.22	2.26	2.26	2.15	2.13	2.13
**C_{dl} (mF cm^{-2})**	2.62	1.29	1.51	1.46	1.75	4.15	1.48
**ECSA**	66.75	35.5	41.1	39.1	46.2	106.15	38.4
**Overpotntial (mV) at 10 mA cm^{-2}**	326	318	307	299	319	280	284
**Overpotntial (mV) at 50 mA cm^{-2}**	424	394	363	363	378	337	326
**Overpotntial (mV) at 100 mA cm^{-2}**	507	457	397	390	423	354	376
**Tafel slope (mV dec^{-1})**	96.6	86.7	71.8	69.3	66.8	59.9	59.5

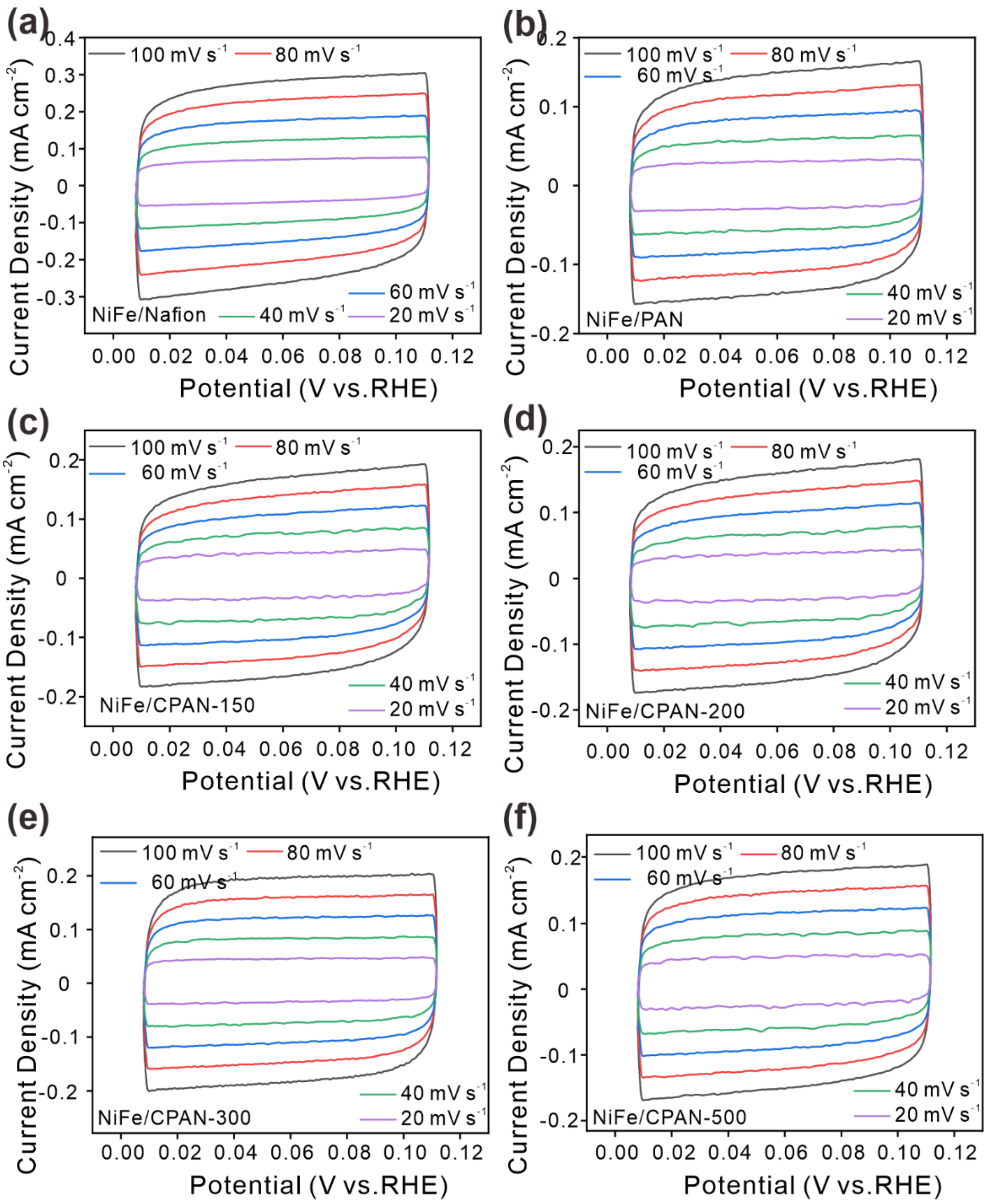

Figure S5. CV curves under various scan rates (scan rates: 20 to 100 mV s^{-1}) of the synthesized electrode materials: (a) NiFe/Nafion, (b) NiFe/PAN, (c) NiFe/CPAN-150, (d) NiFe/CPAN-200, (e) NiFe/CPAN-300, and (f) NiFe/CPAN-500.

Table S2. Comparison of overpotentials for OER catalysts in the literature and in this work.

Catalysts	Overpotential (mV)	Current densities (mA cm^{-2})	Ref
**NiFe (hydr) oxides**	346	10	(1)
**NiFe/W0.3C@NC**	290	10	(2)
**NiFe films**	290	10	(3)
**NiFe/CPAN-400**	280<br>337<br>354	10<br>50<br>100	This work
**NiFe/CNTs-900**	257	10	(4)
**Exfoliated NiFe LDH/CB nanosheets**	220	10	(5)
**NiFe HOF**	250	50	(6)
**$Ni_{78}Fe_{22}$**	222<br>286<br>327	10<br>1000<br>2000	(7)
**FcNiOF**	280<br>284	1000<br>2000	(8)

References

(1) Hashemi, N.; Nandy, S.; Chae, K. H.; Najafpour, M. M. Anodization of a NiFe Foam: An Efficient and Stable Electrode for Oxygen-Evolution Reaction. *ACS Appl. Energy Mater.* **2022**, *5* (9), 11098–11112. https://doi.org/10.1021/acsaem.2c01707.

(2) Jang, E.; Cho, J.; Kim, J.; Kim, J. WC Nanoparticles and NiFe Alloy Co-Encapsulated in N-Doped Carbon Nanocage for Exceptional OER and ORR Bifunctional Electrocatalysis. *Appl. Surf. Sci.* **2024**, *663*, 160201. https://doi.org/10.1016/j.apsusc.2024.160201.

(3) Zhang, M.; Deng, C. Temperature-Dependence of Magnetron Sputtered NiFe Films: Structure, Morphology, Optical, Electrical and OER Catalytic Properties. *Appl. Surf. Sci.* **2024**, *653*, 159317. https://doi.org/10.1016/j.apsusc.2024.159317.

(4) Li, H.; He, Y.; He, T.; Shi, H.; Yu, H.; Ma, X.; Zhang, Y.; Zhang, C.; Wang, S. Facile Fabrication of Activated NiFe Bimetallic NPs Anchored N-Doped CNTs Arrays as Reliable Self-Standing Electrocatalyst for HER and OER. *J. Solid State Chem.* **2020**, *289*, 121498. https://doi.org/10.1016/j.jssc.2020.121498.

(5) Munonde, T. S.; Zheng, H.; Nomngongo, P. N. Ultrasonic Exfoliation of NiFe LDH/C

B Nanosheets for Enhanced Oxygen Evolution Catalysis. *Ultrason. Sonochem.* **2019**, *59*, 104716. https://doi.org/10.1016/j.ultsonch.2019.104716.

(6) Liao, H.; Chen, K.; He, X.; Tong, J.; Liu, X.; Tan, P.; Guo, X.; Pan, J. Metal Hydroxide–Organic Framework Mediated Structural Reengineering Enables Efficient NiFe Interaction for Robust Water Oxidation. *Nano Lett.* **2024**, *24* (48), 15436–15443. https://doi.org/10.1021/acs.nanolett.4c04815.

(7) Nairan, A.; Feng, Z.; Zheng, R.; Khan, U.; Gao, J. Engineering Metallic Alloy Electrode for Robust and Active Water Electrocatalysis with Large Current Density Exceeding 2000 mA Cm^{-2}. *Adv. Mater.* **2024**, *36* (29), 2401448. https://doi.org/10.1002/adma.202401448.

(8) Chen, Y.; Li, Q.; Lin, Y.; Liu, J.; Pan, J.; Hu, J.; Xu, X. Boosting Oxygen Evolution Reaction by FeNi Hydroxide-Organic Framework Electrocatalyst toward Alkaline Water Electrolyzer. *Nat. Commun.* **2024**, *15* (1), 7278. https://doi.org/10.1038/s41467-024-51521-4.
